# Atypical Kawasaki syndrome in COVID-19 infection: a case report of a multisystem inflammatory syndrome in a child (MIS-C)

**DOI:** 10.4314/gmj.v55i2s.10

**Published:** 2021-06

**Authors:** Justice Sylverken, Priscilla Afari, Charles Martyn-Dickens, Sheila A Owusu, Emmanuel Oppong, Francis Akwetey, Ekow Mensah, Haruna Mahama, Sandra K Owusu, Sampson Antwi

**Affiliations:** 1 Child Health Directorate, Komfo Anokye Teaching Hospital, Kumasi, Ghana; 2 Department of Child Health, School of Medicine and Dentistry, College of Health Sciences, Kwame Nkrumah University of Science and Technology, Kumasi, Ghana

**Keywords:** SARS-CoV-2, COVID-19, Children, MIS-C, Kawasaki-like syndrome

## Abstract

The emergence of COVID-19 by a novel severe acute respiratory syndrome coronavirus (SARS-CoV-2) in 2019 has seen evolving data reporting infrequent infection in children and mostly mild disease for children who contract the infection. A severe form of COVID-19 in children recently reported in Europe and North America describes a multisystem inflammation syndrome in children (MIS-C), presenting as toxic-shock-like and Kawasaki-like syndromes. Data on MIS-C in Africa is being documented with recent reports from South Africa and Nigeria in black children, but information on MIS-C in Ghana is yet to be characterized. We report the first case of multisystem inflammatory syndrome in a child who tested PCR positive to SARS-CoV2 in a tertiary hospital in Ghana. The case describes a 10-year-old boy who reported Kawasaki-like syndrome without shock but with moderate respiratory distress requiring supportive acute care without the need for intensive care.

**Funding:**

None declared

## Introduction

The COVID-19 pandemic caused by the SARS-CoV2 affects children relatively less than adults, and milder disease is reported in children^1^,^2^,^3^. However, there are published data from United Kingdom (UK) in mid-April 2020 and data from Italy in May 2020 on clusters of severe form, which presents as toxic-shock-like and or Kawasaki-like syndromes, and a similar large case series in the United States of America (USA). Recent MIS-C data from South Africa and Nigeria revealed comparable findings.^5^,^6^ These reports commonly highlighted the presence of cardiovascular shock, fever and hyper inflammation with multisystem involvement, termed Multisystem inflammation in children .^7^,^8^,^9^ Notably, a finding of Kawasaki-like syndrome in older children and adolescents instead of the usual finding of complete Kawasaki syndrome in children-under-five, is what makes the Kawasaki presentation in the setting of COVID-19 infection atypical.^7^,^10^ The mechanism of Kawasaki syndrome has remained elusive, but viral infection is documented as a likely trigger.^11^,^12^

We report the first case of MIS-C in our hospital, a tertiary hospital in Ghana, based on the CDC and WHO criteria defined as a confirmed case of COVID-19 or likely exposure to COVID-19 in a child with persistent fever, elevated inflammatory markers and presence of single or multi-organ involvement. 13,14 The case definition included six criteria; serious illness leading to hospitalization, age less than 21years, fever (body temperature >38°C) or a report of subjective fever lasting at least 24 hours, laboratory evidence of inflammation multisystem organ involvement (i.e. two systems) and laboratory-confirmed SARS-CoV-2 infection, positive SARS CoV-2 Real-time reverse transcriptase polymerase chain reaction [RT-PCR])

## Case Report

Our patient is a 10-year-old male previously well with no known comorbid conditions, who was referred to the Paediatric Emergency Unit of the Komfo Anokye Teaching Hospital for suspected septicaemia. He had a 7-day history of intermittent high-grade fever. Two days after the onset of the fever, he also experienced neck pain, neck swelling, red eyes, red, cracked lips, and dysphagia. At presentation, he had an episode of vomiting and passage of greenish watery stool, associated with abdominal pain.

On presentation at the emergency unit, the patient was alert, with an initial temperature of 37.2°C, and physical examination revealed bilateral non-exudative conjunctivitis, angular stomatitis, strawberry tongue, solitary cervical lymph node (approximately 1cm) and bilateral swollen feet. These signs raised a high suspicion for Kawasaki syndrome with differentials of COVID-19 infection and infectious mononucleosis. Additionally, he had hepatomegaly (6cm below the costal margin) but no enlarged spleen.

The patient was not in respiratory distress and had no abnormal cardiac findings on clinical examination on initial assessment. However, on day 2 of admission, the patient spiked a temperature of 40.5°C and developed respiratory distress: RR=44/min from baseline of 22/min. The patient was also noted to have flaring of the ala nasae and intercostal retractions, with SpO_2_ of 92% on room air from a baseline of 99%.

Relevant laboratory findings were: mild anaemia (Hb of 9.5g/dl, moderate thrombocytopenia (47x10^9^/L, leukocytosis (20.4x10^9^/L, neutrophilia(17.25x10^9^/L), marginally raised ESR(30mm fall/hr) and acute kidney injury(urea of 29.35 mmol/l and creatinine of 300umol/L. Serum sodium, and potassium were normal. His chest radiograph showed bilateral ground-glass opacities, and there was evidence of pericarditis with mild pericardial effusion on echocardiogram. The patient tested positive to SARS-CoV-2 by RT-PCR on Day 2 of admission.

After the initial assessment, management was supportive, and the patient was nursed in the isolation ward for suspected COVID-19 infection. He was given oxygen support via nasal prongs for three days on account of respiratory distress. Empiric intravenous ceftriaxone was initiated to cover for possible bacterial meningitis owing to his presentation of fever, headache and neck pain. Still, on subsequent reviews, the neck pain was due to an enlarged tonsil, and he had no signs of meningism and remained fully conscious with no seizures. Lumbar puncture was therefore not done and more so due to the thrombocytopenia. Subsequently, we added azithromycin 250mg PO daily for three days based on suspicion of COVID-19. Aspirin, a standard therapy for Kawasaki syndrome, was withheld because he had moderate thrombocytopenia, and IV immunoglobulin was not initiated due to cost.

Other medications given were largely in keeping with the national COVID-19 treatment protocol: Oral Vitamin C 1g daily x 14 days, oral Zinc 20mg daily x 14 days, dexamethasone 4mg daily x 2 days, oral paracetamol 250mg 8 hourly for six days and topical anaesthesia (Bongella gel) for the mucositis. He was discharged after eight days of admission in a stable state with counselling on isolation and infection prevention measures for the family. The patient was passing adequate amounts of urine at the time.

The patient was firstly reviewed a week after being discharged and remained stable. Upon the second review, on Day 30 after the onset of illness, his renal function test had normalized, and LFTs remained normal. Unfortunately, a repeat full blood count test that was requested at the same time never got taken.

The patient had developed an ataxic gait. However, brain CT/MRI, as requested by the paediatric neurologist, couldn't be done because the machine broke down, and caregivers did not report for a subsequent imaging appointment. He was started on physiotherapy and referred for follow-up at the pediatric neurology clinic

## Discussion

While it is not unusual that we are documenting a case of MIS-C in a ten-year-old boy, based on the age group of patients with reported MIS-C cases, it is not typical to see Kawasaki presentation in older children 7,10. With the community spread of COVID-19 in Kumasi, it is not surprising that our patient had no known exposure to an index or suspected case, and he had no recent international or intercity travel.

The presence of fever, red eyes, cracked lips, strawberry tongue, cervical lymphadenopathy and raised inflammatory makers is typical of Kawasaki syndrome. What makes this presentation in our patient and other reported MIS-C cases with Kawasaki-like syndrome atypical is the older age of onset, gastrointestinal symptoms and left ventricular dysfunction.^1^,^7^,^8^,^9^,^15^ Notably, our patient uniquely had pericarditis on echocardiogram with normal left ventricular function. In a multi-centre US MIS-C case report, 137 out of 186 patients had mucocutaneous symptoms.^9^ A south African case series involving 21 children and a case report from Nigeria documented similar findings^5^,^6^. Comparably, while a report from an Italian study showed 30% of cases with conjunctivitis, a New York study revealed 27% of patients with mucosal changes and 57% with conjunctival injection out of 99 MIS-C cases.^8^,^16^ A finding of respiratory and gastrointestinal symptoms in our patient collaborates with published MIS-C case reports.^7^,^8^,^9^,^15^

Laboratory findings in our patient showed positive PCR test to SARS-CoV-2 and raised inflammatory markers similar to published cases.^1^,^7^,^8^,^9^,^15^ Ground glass appearance on chest radiographic findings were not different from other reports. Still, the echocardiography done 13 days after the onset of fever and on day 6 of admission showed pericarditis with minimal pericardial effusion similar to an Italian case series, where 4 out of 10 patients had pericardial effusion.^8^ In contrast, we found no evidence of myocardial inflammation or left ventricular dysfunction, as seen in other reports.^7^,^8^,^9^,^15^ Perhaps, earlier echocardiography or test of cardiac enzymes might have revealed myocarditis as was the case in MIS-C in York State, where 53% of patients had myocarditis. However, another report also suggests that myocarditis may be absent in MIS-C. In multi-centre US data on MIS-C, the authors documented the presence of a coronary aneurysm in 15 out of 186 patients. In contrast, a UK MIS-C report documented left ventricular dysfunction in 7 out of 8 cases. 9,7

In some children with MIS-C, circulatory shock requiring ICU care was noted.^7^,^9^,^16^ Similarly, a Nigerian case report in a 12-year old involved ICU care.^6^ Our patient did not develop shock, but he was managed at the high dependency emergency unit with oxygen support, intravenous maintenance fluids and regular oxygen saturation and vitals monitoring. Some children who tested positive for other respiratory viruses like influenza and adenovirus infection had severe disease and required ICU care in the UK study.^7^ As the mechanism of MIS-C is still evolving, it is unclear whether concurrent influenza or adenoviral infection may have influenced the need for ICU care in our patient, especially when earlier studies of severe human coronaviruses infection children before the COVID-19 pandemic were rather seen in children under three or those with heart disease, and not necessarily respiratory syncytial virus coinfection^1^. Moreover, while some infectious disease experts postulate that genetic factors may play a role in developing severe MIS-C, some authors suggest an interplay between delayed hyperimmune response and severe MIS-C following a mild COVID-19 may have implications for future vaccine development.^17^,^18^ We withheld giving aspirin, a standard therapy for Kawasaki disease, on account of moderate thrombocytopenia and cost, respectively^19^. The cost of IV immunoglobulin is prohibitive and similar to the Nigerian case; we did not give it because caregivers could not afford it.^6^

## Conclusion

Each case of MIS-C, though criterion-based, may vary. A Kawasaki-like syndrome without shock and echocardiographic findings of pericarditis in our case adds to the existing body of data on the different characteristics of MIS-C in children.
